# Supplementary figures and images for: Role of Toxoplasma gondii Chloroquine Resistance Transporter in Bradyzoite Viability and Digestive Vacuole Maintenance
Source: mBio. 2019 Aug 6;10(4):e01324-19. doi: 10.1128/mBio.01324-19 (PMC6686041; doi:10.1128/mBio.01324-19)

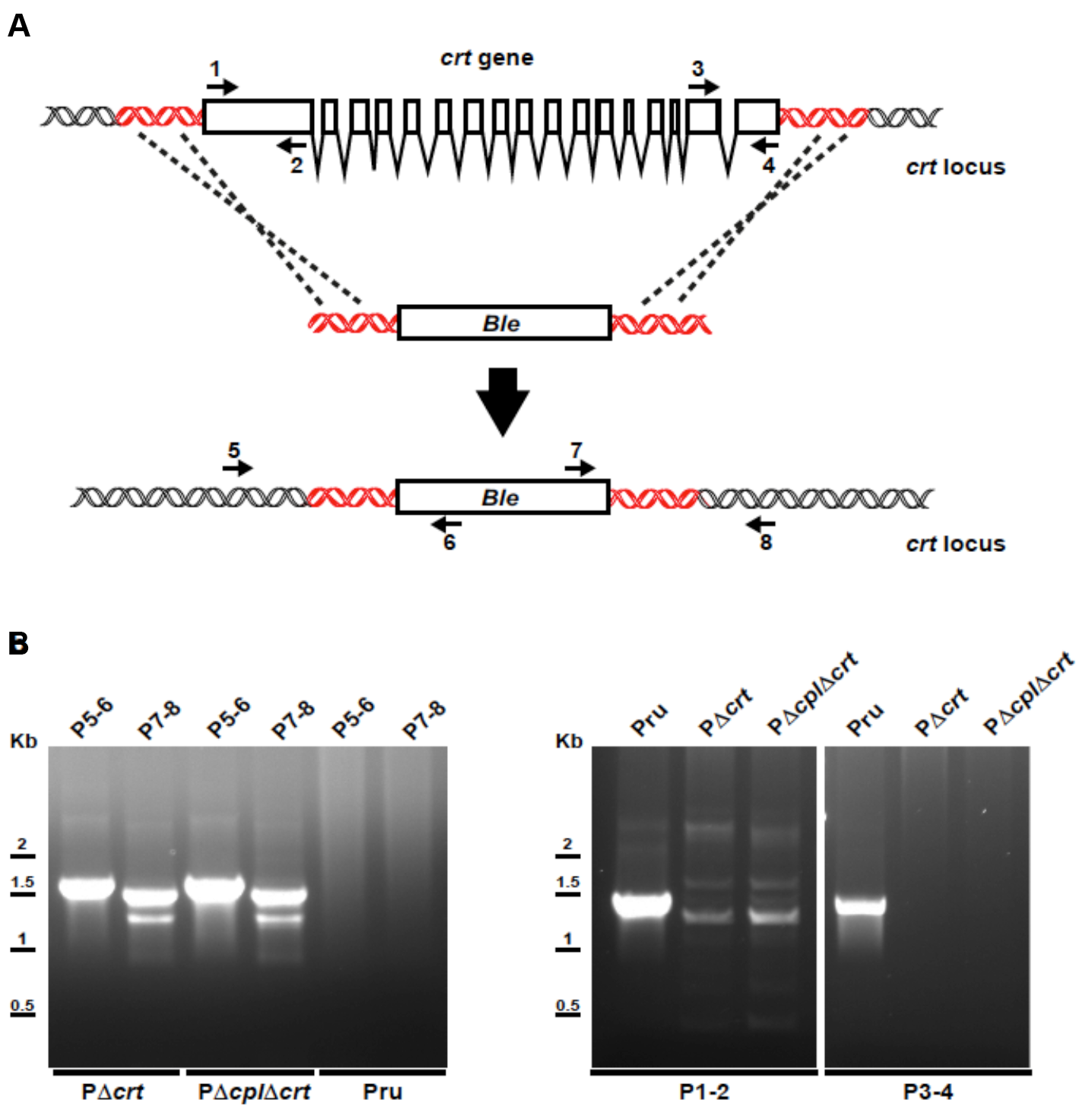

Supplement: FIG S1 [file mBio.01324-19-sf001.tif]

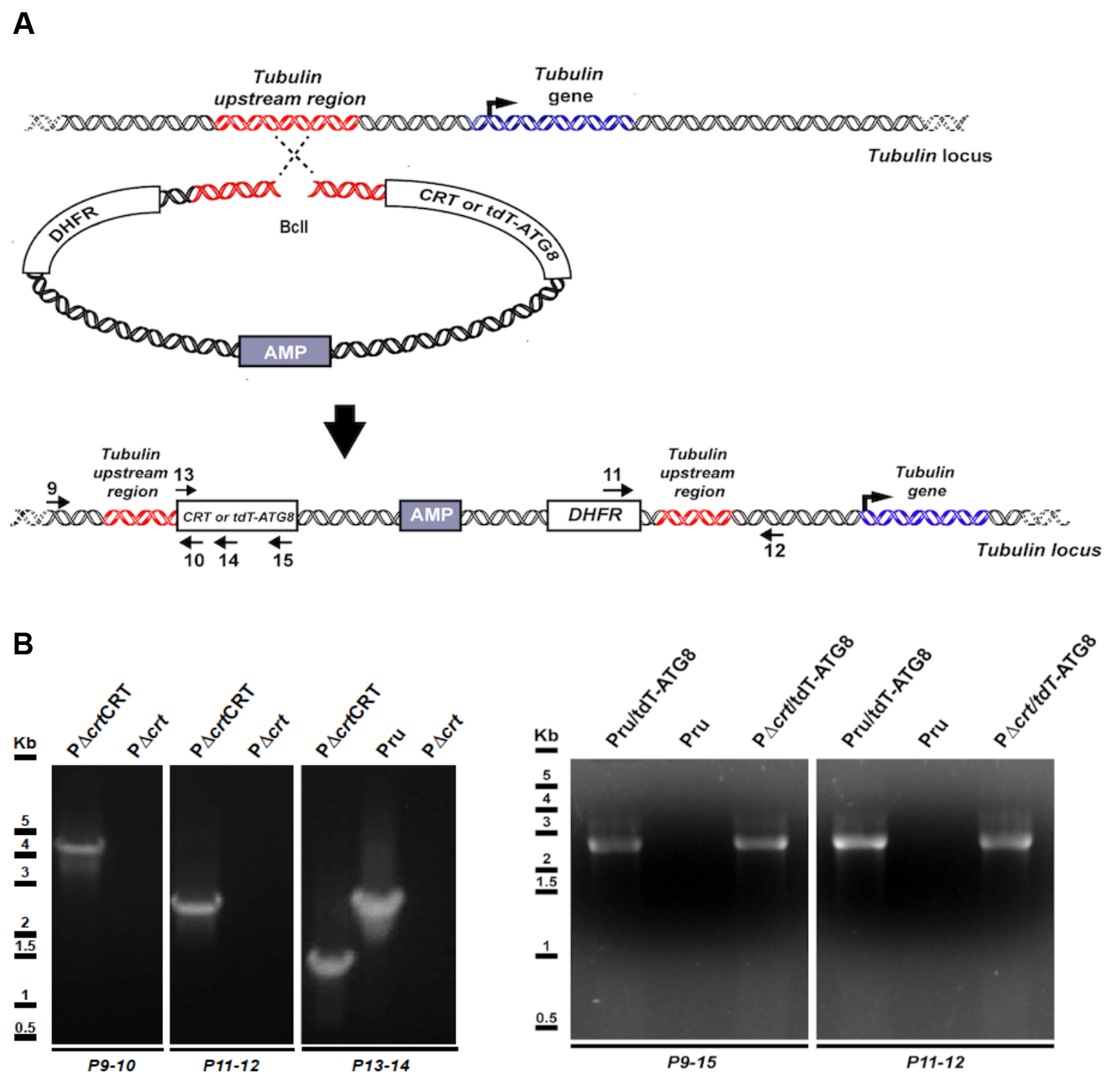

Supplement: FIG S2 [file mBio.01324-19-sf002.tif]

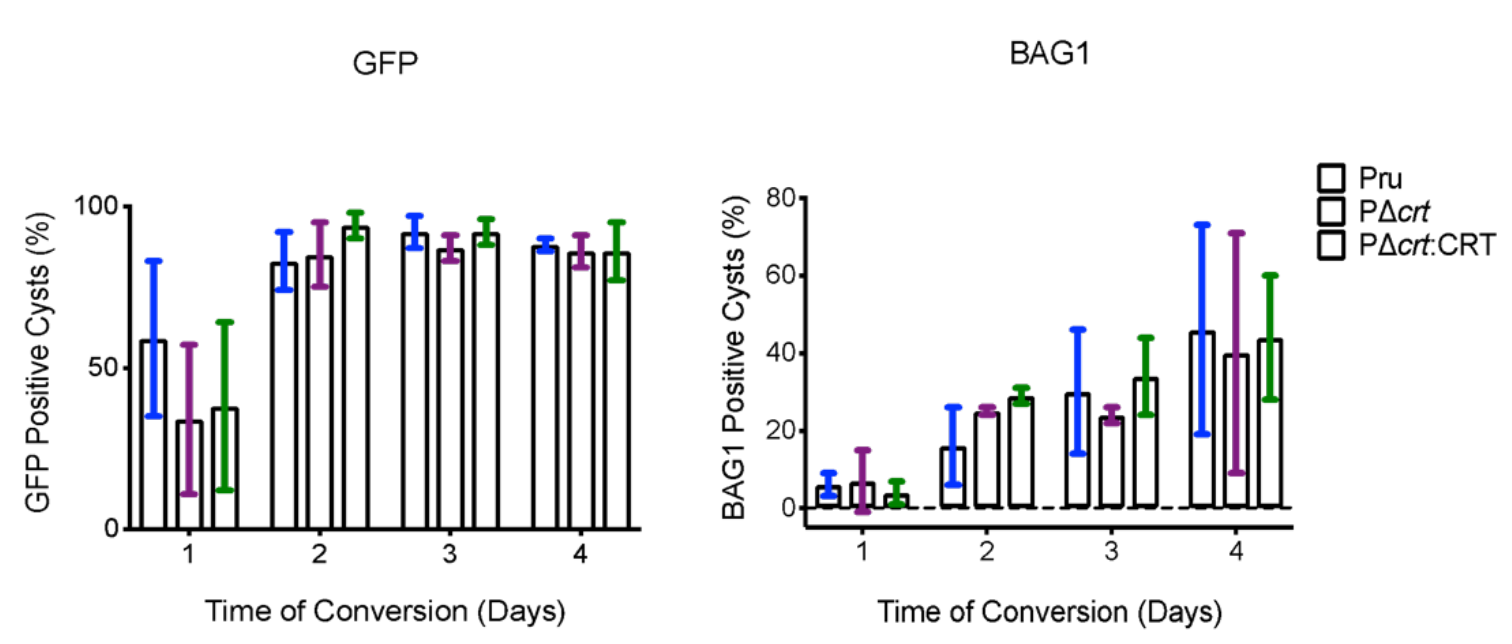

Supplement: FIG S3 [file mBio.01324-19-sf003.tif]

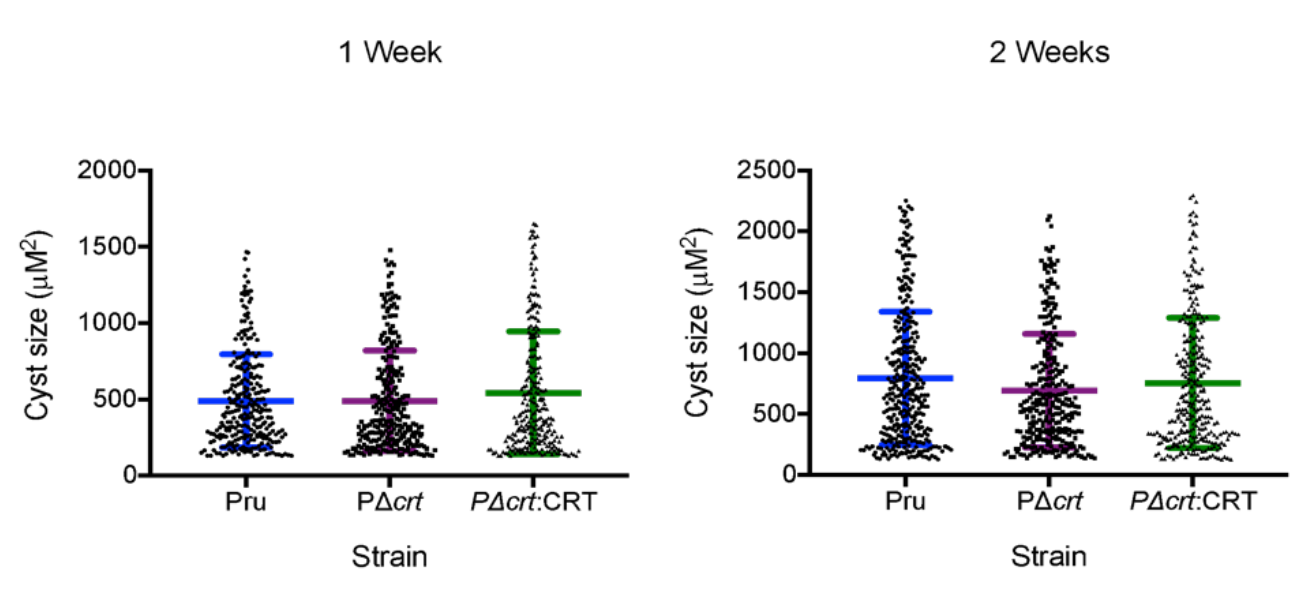

Supplement: FIG S4 [file mBio.01324-19-sf004.tif]

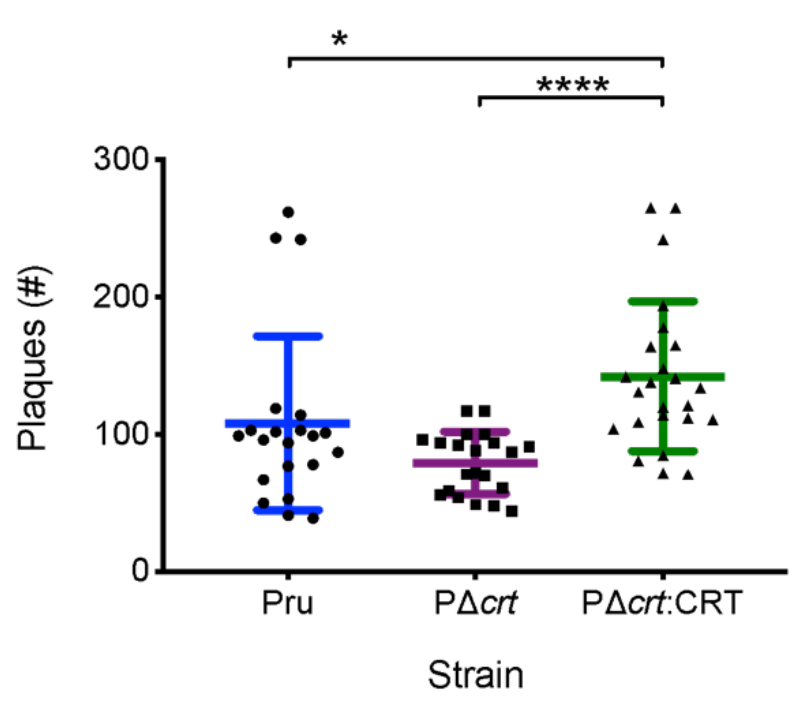

Supplement: FIG S5 [file mBio.01324-19-sf005.tif]
