# Supplementary material for: Role of Toxoplasma gondii Chloroquine Resistance Transporter in Bradyzoite Viability and Digestive Vacuole Maintenance
Source: mBio. 2019 Aug 6;10(4):e01324-19. doi: 10.1128/mBio.01324-19 (PMC6686041; doi:10.1128/mBio.01324-19)
Supplement: TABLE S1 [file mBio.01324-19-st001.docx]

| **Primer Name** | **Forward Primer**  **(5’- 3’)** | **Reverse Primer**  **(5’-3’)** | **Product**  **Size (bp)** |
| --- | --- | --- | --- |
| **Deletion of CRT (Fig. S1)** | | | |
| 5’ ARM of CRT; upstream of BLE with *Xho*I/ *Hind*III sites | CCCCCTCGAGGTGCACCGTTTGACACATCTGATTGTG | TTCGAAGCTTGTCGCGCTAGCACAGCTGTGAGAGACTC | 1530 |
| 3’ ARM of CRT; downstream of BLE with *BamH*I/*Not*I sites | GGGGGATCCATCGCCGAACAGAGTTGGTGGCTACGAG | GGTGGCGGCCGCGAGAGATTACCCTACTGCGCATCCGTAC | 1440 |
| P1/P2 | GCGCCTTCGCAGCAGACTGATGTTCGAAG | GTGAGTCCGGAGCCTGAGAGGTCCTTC | 1487 |
| P3/P4 | GAAGAGCAGATCGGGACCATTTTCGTC | CCCGTGCCTCCAGCGAAGCCTGTCTCTTC | 1470 |
| P5/P6 | GCTCTGTAGGACTTGCATCACCAACGAG | GCGTGTCGCATACGAGCACCAGTACTAC | 1647 |
| P7/P8 | CAGTTGTTTTAGTCGAACCGGTTAACA | GGAGCAGCGGATGCAAGCCTTTTTCTGTG | 1542 |
| **Generation of CRT Complement (Fig. S2)** | | | |
| P9/P14 | CAGCACGTGACTCGATGTTTACCGCTGTC | CTCCGGCGTAGTCGGTGTACAAGGAG | 4546 |
| P11/P12 | ATGGAGATGGCTGTCTAGTTAATTAATC | CCTGGCCGACGTGGATGCTGATAACCTC | 2575 |
| P13/14 | CACAGACTGCTTGTGTACCTCCGTG | GTATGCACAGCACCGATGATGGCCATC | 1490 (cDNA), 2837 (gDNA) |
| **Generation of tdTomato-Atg8 (Fig. S2)** | | | |
| P9/15 | CAG CAC GTG ACT CGA TGT TTA CCG CTG TC | CCA TGC GCA CCT TGA AGC GCA TGA ACT C | 2842 |
| P11/12 | CTA TCA GTTG TTT AGT CGA ACC GGT TAA C | CTG GCC GAC GTG GAT AAC CTC | 2869 |
| **Parasite burden qPCR (Fig. 3)** | | | |
| **Tox9/11** | AGG AGA GAT ATC AGG ACT GTA G | GCG TCG TCT CGT CTA GAT CG |  |
| **qPCR/Plaque assay (Fig. 2)** | | | |
| **⍺-TUB** | GCG TCT TCT TGG ATT TGG AG | TGG AGA CCA GTG CAG TTG TC |  |
